# Editorial: ENPER2024 - three decades of endomembrane research

**DOI:** 10.3389/fpls.2026.1849403

**Published:** 2026-04-22

**Authors:** Gian Pietro Di Sansebastiano, Cláudia Pereira

**Affiliations:** 1 DiSTeBA (Department of Biological and Environmental Sciences and Technologies), University of Salento, Lecce, Italy; 2 GreenUPorto – Sustainable Agrifood Production Research Centre and INOV4AGRO - Institute for Innovation, Training and Sustainability of Agrifood Production & Department of Biology, Faculty of Sciences of University of Porto, Porto, Portugal

**Keywords:** ENPER research community, membrane dynamics, organelle biogenesis, plant endomembrane system, plant signalling and stress responses, vesicle trafficking

The plant endomembrane system is a highly dynamic network of organelles and trafficking pathways that coordinate protein sorting, membrane remodelling, and cellular signalling. Over the past decades, advances in imaging, molecular genetics, and biochemical approaches have significantly expanded our understanding of endomembrane organization and function. Nevertheless, fundamental questions remain regarding how trafficking pathways are regulated and how they contribute to plant development and environmental responses.

Since its establishment in 1996, the European Network for Plant Endomembrane Research (ENPER) has provided a unique forum for researchers working on plant membrane biology. The ENPER meetings have played an important role in fostering collaboration and discussion across disciplines, bringing together scientists studying vesicle trafficking, organelle biogenesis, membrane signalling, and plant–microbe interactions. As one of the longest-running conferences dedicated to plant membrane biology, ENPER provides a platform where researchers - from early-career scientists to established researchers - share new discoveries and emerging concepts in endomembrane trafficking with a collaborative approach that contributed to accelerate progresses in the past decades. The ENPER2024 meeting, held in Porto, Portugal, continued this tradition by bringing together researchers working on diverse aspects of membrane dynamics, protein transport, and organelle communication.

This Research Topic, *ENPER2024 – Three Decades of Endomembrane Research*, compiles contributions that reflect both the diversity of approaches and the continuing evolution of the field. The articles collected here highlight recent advances in understanding trafficking mechanisms, vacuolar and endosomal functions, and the interplay between membrane systems and plant signalling pathways.

Different contributions explore the molecular machinery governing vesicle trafficking and membrane organization with an impact of cell biology of all eukaryotes and not only plants. In the review *“The clues offered by SNAREs on the vacuoles of plants and animals”* Barozzi et al. examine the conserved and divergent roles of SNARE proteins in mediating membrane fusion events. By comparing plant and animal vacuolar systems, the authors emphasize how studies in different organisms can illuminate the evolutionary conservation of trafficking mechanisms while also revealing plant-specific adaptations. Their synthesis highlights the importance of SNARE complexes in maintaining vacuolar identity and regulating membrane fusion processes essential for cellular homeostasis. The importance of trafficking components in plant development is further illustrated by the original research article *“Exocyst subunits EXO70B1 and B2 contribute to stomatal dynamics and cell wall modifications”* Drs et al. investigate the roles of specific exocyst subunits in stomatal function, providing evidence that EXO70B1 and EXO70B2 contribute to the regulation of cell wall remodelling and guard cell dynamics. These findings underscore the complex roles of the exocyst complex in coordinating targeted secretion and cellular morphogenesis, particularly in specialized cell types such as guard cells.

Membrane dynamics are also central to organelle biogenesis and differentiation. In the study *“The fate and function of the Arabidopsis Class I formin AtFH1 during central vacuole biogenesis in the rhizodermis”* Kočová et al. explore how the actin-associated protein AtFH1 contributes to vacuole formation. Their work reveals a dynamic interplay between cytoskeletal elements and membrane remodelling processes during the development of the central vacuole, an organelle essential for plant cell expansion and physiological regulation.

Beyond developmental processes, the endomembrane system also plays a critical role in plant responses to environmental cues and biotic interactions. The mini-review *“Salicylic acid: new pathways arising?”* by Müller and Scheuring revisits the emerging connections between salicylic acid signalling and membrane trafficking pathways. The authors discuss recent evidence suggesting that hormone signalling and endomembrane dynamics are closely connected, opening new perspectives on how intracellular transport processes influence immune responses and stress signalling. In the mini-review *“Pathogen effector tactics to suppress plant endomembrane system”* In et al summarize current knowledge on how pathogen effectors target host trafficking pathways to promote infection. By interfering with vesicle trafficking and membrane identity, pathogens can disrupt host immune responses and cellular organization. This review highlights the endomembrane system as both a critical component of plant defence and a vulnerable target exploited by pathogens.

Taken together, the contributions assembled in this Research Topic reflect the broad but central role of membrane trafficking in plant cell biology. From vesicle fusion machinery and cytoskeleton–membrane interactions to hormone signalling and pathogen interference, the endomembrane system emerges as a key integrator of cellular processes. For a long period in which research was focused on basic traffic mechanisms and complexity of the plant cell had to be fully recognized, we assist now to the emerging of the intricate relation between endomembranes and cellular responses to all possible external stimuli. The studies published in this Research Topic as the research conducted in the ENPER community in general, also illustrate how combining different methodological approaches, including molecular genetics, cell biology, and comparative analysis, continues to advance our understanding of intracellular transport mechanisms. Importantly, the Research Topic reflects the collaborative and interdisciplinary spirit that has characterized the ENPER community for nearly three decades. The ENPER meetings have consistently fostered interactions between laboratories working on complementary aspects of membrane biology, facilitating the exchange of ideas and the development of new conceptual frameworks. As new technologies - such as advanced live-cell imaging, quantitative proteomics, and computational modelling - become increasingly accessible, the field is well positioned to address longstanding questions regarding membrane organization and dynamics.

Looking forward, several challenges remain. Understanding how trafficking pathways are coordinated at the systems level, how membrane identity is established and maintained, and how organelles communicate during development and stress responses are among the key questions that will shape future research. Addressing these questions will require continued collaboration across disciplines and the integration of experimental and theoretical approaches.

The contributions gathered in *ENPER2024 – Three Decades of Endomembrane Research* provide valuable insights into these topics and highlight the vitality of the plant endomembrane research community. We thank all authors, reviewers, and editors who contributed to this Research Topic and hope that the work presented here will stimulate further research and collaboration within this dynamic field.

